# Canadian supportive care recommendations for the management of neutropenia in patients with cancer

**DOI:** 10.3747/co.2008.198

**Published:** 2008-01

**Authors:** C.T. Kouroukis, S. Chia, S. Verma, D. Robson, C. Desbiens, C. Cripps, J. Mikhael

**Affiliations:** * Juravinski Cancer Centre, Hamilton, ON; † BC Cancer Agency, Vancouver, BC; ‡ Sunnybrook Health Sciences Centre–Odette Cancer Centre, Toronto, ON; § Centre Hospitalier Affilie de Quebec–Hôpital du St-Sacrement, Quebec City, QC; || Ottawa Hospital Regional Cancer Centre, Ottawa, ON; # Princess Margaret Hospital, Toronto, ON

**Keywords:** Canadian recommendations, neutropenia, febrile neutropenia, supportive care, colony-stimulating factors, chemotherapy-induced neutropenia, safety

## Abstract

Hematologic toxicities of cancer chemotherapy are common and often limit the ability to provide treatment in a timely and dose-intensive manner. These limitations may be of utmost importance in the adjuvant and curative intent settings. Hematologic toxicities may result in febrile neutropenia, infections, fatigue, and bleeding, all of which may lead to additional complications and prolonged hospitalization. The older cancer patient and patients with significant comorbidities may be at highest risk of neutropenic complications. Colony-stimulating factors (csfs) such as filgrastim and pegfilgrastim can effectively attenuate most of the neutropenic consequences of chemotherapy, improve the ability to continue chemotherapy on the planned schedule, and minimize the risk of febrile neutropenia and infectious morbidity and mortality. The present consensus statement reviews the use of csfs in the management of neutropenia in patients with cancer and sets out specific recommendations based on published international guidelines tailored to the specifics of the Canadian practice landscape. We review existing international guidelines, the indications for primary and secondary prophylaxis, the importance of maintaining dose intensity, and the use of csfs in leukemia, stem-cell transplantation, and radiotherapy. Specific disease-related recommendations are provided related to breast cancer, non-Hodgkin lymphoma, lung cancer, and gastrointestinal cancer. Finally, csf dosing and schedules, duration of therapy, and associated acute and potential chronic toxicities are examined.

## 1. INTRODUCTION

Advances in cancer treatment have led to the development and administration of more-complex chemotherapy regimens in a wider spectrum of cancer patients, often resulting in increases in hematologic toxicities. Also, as the population ages and demographics shift, greater numbers of older adults, some with significant comorbidities, are being considered for chemotherapy that may result in significant toxicity.

Agents to attenuate hematologic toxicities have been in widespread use, particularly in primary and secondary prevention of the neutropenia and febrile neutropenia (fn) associated with chemotherapy and stem-cell transplantation (sct). In cancer patients, fn has been shown to have a significant impact on patient outcomes and on the health care system 1. The potential benefits of colony-stimulating factors (csfs) such as granulocyte colony–stimulating factor (g-csf) must be measured against their cost and potential toxicities.

The consensus statement presented here was prepared to summarize, from the Canadian perspective, current guidelines on the use of csfs, taking into account the available evidence and recently updated international guidelines 2–4. The present paper reviews the updated guidelines, recommendations for primary and secondary prophylaxis, and the use of growth factors in leukemia, sct, and radiotherapy. In addition, disease-specific recommendations are made for breast cancer, lymphoma, lung cancer, and gastrointestinal cancer. Finally, the latest safety information regarding the use of growth factors is discussed.

## 2. SUMMARY OF EXISTING GUIDELINES

The American Society of Clinical Oncology (asco), the European Organization for Research and Treatment of Cancer (eortc), and the National Comprehensive Cancer Network (nccn) have all published guidelines for the use of csfs in patients with cancer 2–4 (Table I).

The 2005 asco guidelines were updated 2 from the 2000 version; their evidence base included medline and Cochrane Library searches up to and including September 2005. The asco guidelines endorse the importance of preventing fn as a clinical outcome, regardless of other factors, particularly when the fn rate associated with treatment is at least 20% and no other equally efficacious regimen that would not require csfs is available. Primary prophylaxis with csfs is recommended for fn prevention in patients at higher risk based on age, medical history, disease characteristics, and myelotoxicity of the chemotherapy regimen. Dose-dense chemotherapy requiring csfs is recommended only when clearly supported by the available evidence or as part of a clinical trial. Prophylactic csf is suggested for older patients (65 years of age or older) with aggressive-histology lymphoma treated with curative intent. Secondary prophylaxis is recommended for patients who experience a neutropenic complication associated with an earlier chemotherapy cycle and in whom a reduced dose in a subsequent cycle could compromise disease-free survival (dfs), overall survival (os), or another treatment outcome.

In 2006, eortc published its guidelines for the use of csfs in patients with cancer and chemotherapy-induced neutropenia. Those guidelines were based on literature published from 1996 through September 2005 3. In 2003, the eortc Cancer in the Elderly Task Force had published guidelines regarding the use of csfs in elderly patients with cancer 5. The eortc guidelines recommend prophylactic csfs when the fn rate of the proposed treatment is 20% or more. In the case of regimens with fn rates of between 10% and 20%, the decision to use csfs should be based on patient-related risk factors, such as older age (over 65 years of age), advanced stage of disease, previous fn episodes, and lack of antibiotic prophylaxis. As do the asco guidelines, the eortc guidelines recommend csfs when a reduction in chemotherapy is associated with poorer prognosis and when dose-dense regimens associated with a clinical and survival benefit are being used.

The updated nccn guidelines for the management of chemotherapy-induced neutropenia 4 are based on a panel review of available evidence. The nccn guidelines recommend routine csf use to reduce the risk of fn, the risk of hospitalization, and the use of intravenous antibiotics in patients treated with a regimen associated with a 20% risk of fn (category 1 evidence). That recommendation encompasses patients receiving curative or adjuvant treatment and treatment to prolong survival or to improve quality of life. For patients being treated with regimens associated with a 10%–20% risk of fn, consideration should be given to using a csf in high-risk patients, but csfs should not be used in low-risk patients (those with a less-than-10% risk of fn), unless a specific patient is at significant risk of serious consequences of fn and that patient is being treated with curative or adjuvant intent.

Filgrastim (Neupogen: Amgen, Thousand Oaks, CA, U.S.A.), pegfilgrastim (Neulasta: Amgen), and ancestim (Stemgen: Amgen) are the only csfs approved for use in Canada.

## 3. GUIDELINES FOR PROPHYLAXIS

### 3.1 Primary Prophylaxis

**FIGURE 1:**
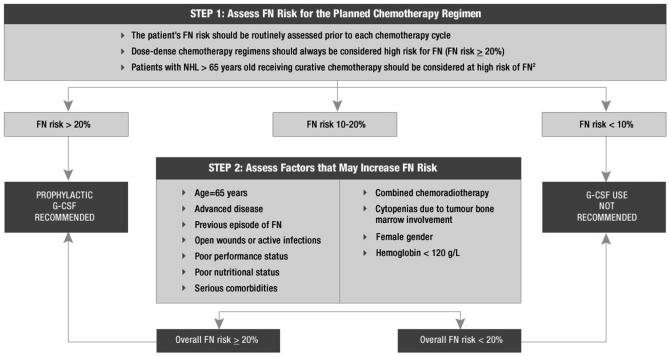
Combined European Organization for Research and Treatment of Cancer 3 and American Society of Clinical Oncology 2 algorithm for primary prophylaxis with granulocyte colony–stimulating factor (g-csf). fn = febrile neutropenia; nhl = non-Hodgkin lymphoma.

Updated international guidelines 2,3 have suggested broadening the indication for csf use for primary prophylaxis in patients with solid tumours and hematologic malignancies alike. The upfront use of g-csfs is suggested with the use of dose-dense chemotherapy in some patients with breast cancer 6 and hematologic malignancies 7. Figure 1 shows a combined eortc and asco algorithm (combined interpretation of the 2006 g-csf guidelines of asco 2 and eortc 3) for primary g-csf prophylaxis.

Based on the asco and eortc updates, the threshold to recommend primary prophylaxis with g-csfs was reduced from a 40% to a 20% risk of fn. The initial estimate of 40% had been calculated from a pharmacoeconomic study 8 based on the results of a randomized study using g-csf in patients with small-cell lung cancer (sclc) 9. The pharmacoeconomic analysis indicated that prophylactic use of g-csfs was cost-effective when the fn rate was at least 40%. The current lower value of 20% risk was arrived at using clinical rather than economic evaluation. Additionally, more recent studies have indicated that g-csfs can dramatically reduce the fn rate even in patients with a baseline fn risk of about 20% 10,11.

The evaluation of neutropenia risk has been summarized for several types of chemotherapy regimens (see the eortc guidelines’ Table 4 or the asco guidelines’ Table 1 for a complete list). Although the relevant data were taken from clinical trials, it is important to realize that the risk of fn in practice could be substantially higher, particularly if patients are older or have comorbidities that may render them ineligible for most clinical trials and increase their risk of complications 1,5. The asco and eortc guidelines have both highlighted the higher risk of fn and infectious complications in older cancer patients.

In a systematic review of randomized trials published up to December 2006 that tested primary prophylaxis with csfs in patients with solid tumours or lymphoma, significant improvements were noted in infection-related mortality, early mortality, and fn 12. Patients receiving csfs experienced more bone pain and a higher average relative dose intensity (di). The hospitalization rate and cancer-related survival data were insufficient for a complete analysis.

In a 2004 Cochrane review that included studies up to August 2003, use of csfs as primary prophylaxis in patients with malignant lymphoma receiving conventional chemotherapy was associated with a reduction in the risk of severe neutropenia, fn, and infection. No evidence of benefit was observed for a reduction in the number of patients receiving intravenous antibiotics, a lower infection-related mortality, or any improvement in tumour response, freedom from treatment failure, or os 13.

No published Canadian economic models have investigated the cost effectiveness of g-csfs for patients with at least a 20% risk of fn, and economic evaluations from other jurisdictions may not be applicable to Canadian practice 14. The asco recommendations emphasize that the decision to use g-csfs to prevent fn should be based on clinical data rather than on economics, looking at evidence of reduction in infection-related endpoints.

A number of models that were developed to assist in predicting neutropenic complications from chemotherapy have previously been summarized 15–17. In one example, targeted filgrastim therapy based on the nadir absolute neutrophil count (anc) in the first cycle of adjuvant treatment for breast cancer resulted in fewer hospitalizations, but no clinical outcome advantages in survival or quality of life were observed 18,19.

#### 3.1.1 Summary of Guidelines for Primary Prophylaxis

- The use of csfs is recommended if the treatment being contemplated is associated with a fn rate of at least 20%, particularly in the curative or adjuvant setting.
- The use of csfs is recommended when risk factors that may increase the toxicities of chemotherapy— such as older age (≥65 years), comorbidities, and previous neutropenic complications—are present.
- When patients are being treated with regimens associated with a 10%–20% risk of fn, clinical judgment should be applied regarding the benefits of csfs, based on clinical, laboratory, patient risk, and disease factors.

### 3.2 Secondary Prophylaxis

When maintaining chemotherapy di is important, g-csfs are recommended for patients who have already experienced a neutropenic complication (for example, fn or neutropenia) resulting in a treatment delay. Maintaining di in such situations minimizes treatment delay and infectious morbidity with the intent of avoiding compromise to cancer-related survival. These criteria are expected to apply best to patients receiving curative treatment who have already experienced a significant neutropenic event. In palliative therapy, less myelotoxic regimens or flexibility in the chemotherapy schedule to avoid significant neutropenic events is preferable. Because much of the recent evidence on the use of g-csfs is disease-specific, disease-specific situations [breast cancer, gastrointestinal cancer, lung cancer, and non- Hodgkin lymphoma (nhl)] are discussed later in this paper.

As noted in the asco guidelines, no prospective studies have specifically evaluated the efficacy of secondary prophylaxis. Development of fn would be considered a significant neutropenic event worthy of future g-csf prophylaxis.

#### 3.2.1 G-CSF Use During FN

One randomized study 20 and two systematic reviews 21,22 addressed the issue of csf use during fn. Although benefits were observed in terms of shorter duration of neutropenia, shorter hospitalizations, and perhaps less infectious burden, no differences in survival were seen. The authors felt that the use of g-csfs during fn would be reasonable if the patient was experiencing a complicated fn episode—for example, pneumonia, multi-organ dysfunction, or hypotension.

#### 3.2.2 Summary of Guidelines for Secondary Prophylaxis

- In patients with a previous neutropenic complication, csfs should be used provided that the alternative of dose reduction may impair tumour response, survival, or treatment outcome.
- Use of a csf during fn should be reserved for patients experiencing a complicated fn episode (for example, pneumonia, multi-organ dysfunction, hypotension).

## 4. GUIDELINES FOR MAINTAINING DI

The clinical benefits of maintaining di are perhaps best demonstrated in adjuvant chemotherapy trials in early-stage breast cancer. The concept of di is defined as the amount of drug delivered per unit time (for example, milligrams per square meter delivered per week or per cycle), and its impact on breast cancer outcomes has been the primary hypothesis in several prospective randomized trials. The French Adjuvant Study Group 05 trial compared high and low (50%) dis of epirubicin (E_100_ vs. E_50_ every 21 days) in a combination containing 5-fluorouracil (5-fu), epirubicin, and cyclophosphamide (fec) 23. The higher di arm yielded significant improvements in dfs and os. Similarly, the Cancer and Leukemia Group B 8541 trial compared high, intermediate, and low dis of doxorubicin, in a combination of cyclophosphamide, doxorubicin, and 5-fu 24. At high and intermediate dis, women experienced significantly improved dfs and os over those experienced by women in the low di group. Those two trials demonstrated that, within the standard anthracycline dose range, a threshold effect exists, meaning that adjuvant chemotherapy delivered using a suboptimal di or lower cumulative anthracycline dose (or both) is less efficacious. To maximally improve survival for women with early-stage breast cancer, a critical (“threshold”) di or cumulative anthracycline dose (or both) must be reached.

Other than 50% or lower, the exact threshold reduction in di that adversely affects clinical outcomes remains controversial. An analysis of the pivotal Milan trial that used classical cyclophosphamide, methotrexate, and 5-fu suggested that women who received less than 85% of the scheduled dose had worse clinical outcomes after 20 years of follow-up 25,26. In addition, women who received less than 65% of the scheduled dose did no better than women treated with surgery alone. Conversely, retrospective data from larger cohorts of women treated with classical or intravenous cyclophosphamide, methotrexate, and 5-fu have failed to demonstrate a statistically significant correlation between chemotherapy di and clinical outcome 27,28.

Reduced di of adjuvant chemotherapy because of toxicity or poor treatment tolerance in primary breast cancer is a common occurrence 29. In a study of community practices across the United States involving almost 20,000 women with early-stage breast cancer treated with adjuvant chemotherapy, 55.5% of patients received a di below 85%. In a similar study of approximately 4500 patients with aggressive nhl, slightly more than half of all patients (53%) received a relative di below 85% 30.

### 4.1 Summary of Guidelines for CSFs in Maintaining DI

- The cumulative data suggest that reduced di is a common occurrence in the adjuvant systemic therapy of early-stage breast cancer and in the curative treatment of aggressive nhl.
- Evidence suggests that a minimum di is required to maximize the benefit of chemotherapy; however, the exact threshold remains to be defined.
- Therefore, when deciding to use a csf, di should be considered, because csf administration may allow for a more optimal dose of chemotherapy to be given.

## 5. GUIDELINES FOR SPECIFIC SETTINGS

### 5.1 Acute Leukemia

Colony-stimulating factors have been studied extensively in acute myeloid (aml) and lymphoblastic (all) leukemia, principally because the chemotherapeutic regimens used are highly myelosuppressive, resulting in a high rate of morbidity and mortality attributable to infection. Although the clinical trials differ, various conclusions can be drawn from the existing data:

- In patients completing induction and consolidation chemotherapy for aml, csfs reduce the duration of neutropenia, but do not affect treatment-related mortality or os 2. The effect may be more pronounced during consolidation therapy.
- Long-term data on the use of csfs in leukemia demonstrate no adverse effect on disease status or patient safety 31.
- Cost analyses (in the United States) suggest that the use of csfs is cost effective in adult aml and all 32.
- When used as an adjunct in treatment of adult all, csfs reduce the incidence of severe infections 33,34.
- Colony-stimulating factors may be beneficial when used as “priming” therapy to enhance chemotherapy in patients with aml 35.

#### 5.1.1 Summary of Guidelines for CSFs in Acute Leukemia

- Colony-stimulating factors should be considered in patients with aml completing induction or consolidation chemotherapy who experience neutropenia.
- Colony-stimulating factors should be considered in patients undergoing chemotherapy for all who experience neutropenia.
- In patients with aml, csfs as priming therapy concurrently with chemotherapy may be useful, but cannot be considered routine at the present time.

### 5.2 Stem-Cell Transplantation

Colony-stimulating factors—both g-csf and granulocyte –macrophage csf—are frequently used during autologous and allogeneic hematopoietic sct. Pre-transplant, csfs are used to assist in the mobilization of stem cells from the marrow for peripheral collection. Post-transplant, they are used to reduce infection, shorten hospitalization, and possibly reduce costs.

#### 5.2.1 Mobilization

Growth factors are used in both autologous and allogeneic transplantation mobilization. Repeated studies have validated this collection approach and confirm its superiority over traditional bone-marrow harvest in yielding a better product that enhances engraftment and reduces graft-versus-host disease (gvhd) 36.

When used in combination with chemotherapy or alone in high doses, csfs promote enhanced mobilization 37,38. Among the various regimens tested, the one most commonly used is g-csf 10 μg/kg daily for 7–10 days before apheresis, with or without chemotherapy (that is, high-dose cyclophosphamide). Pegfilgrastim, although not yet approved for this indication, has showed promise 39.

Another agent, ancestim (also known as “stem-cell factor”), has been used to mobilize stem cells and may even be more effective than g-csf alone 40. Ancestim is generally recommended only in patients who do not successfully mobilize with a g-csf–based mobilization strategy 41.

#### 5.2.2 Post SCT

Data from many randomized studies have showed benefit with the use of csfs in sct, but the magnitude of that benefit in yielding clinically important effects has been questioned. A recent Canadian meta-analysis 42 evaluated the use of csfs post-transplant and revealed that csfs

- reduce the risk of documented infection with a risk ratio (rr) of 0.87 [95% confidence interval (ci): 0.76 to 1.0; *p* = 0.05]. The absolute risk reduction was 8%, and the number needed to treat to prevent 1 infection was 13. In allogeneic sct, the consequence may be reduced infection-related mortality.
- reduce the time to neutrophil recovery and to platelet recovery to 50 × 10^9^/L (*p* = 0.02), but not to recovery to 20 × 10^9^/L.
- reduce hospitalization by 3 days (*p* < 0.00001).
- reduce the duration of parenteral antibiotics (*p* = 0.02).
- produce no differences in acute or chronic gvhd, treatment-related mortality, or os.

The heterogeneity of the available studies has left the potential cost–benefit with the use of csfs unclear. However, to date, more studies than not have suggested a positive benefit. Results from the recent Canadian meta-analysis are consistent with other published studies that have demonstrated a benefit in infection reduction but not in os 13,43,44.

The results from an analysis of a European database raised concerns about the potential increase in gvhd in patients receiving csfs 45. However, a long-term evaluation of data from the International Bone Marrow Transplant Registry on the use of csfs in more than 500 patients treated with allogeneic sct demonstrated no long-term benefit or disadvantage with regard to acute or chronic gvhd and os 46.

#### 5.2.3 Summary of Guidelines for G-CSF in SCT

- For mobilization, 5–10 μg/kg daily can be used for 7–10 days before apheresis, with or without chemotherapy.
- Post transplant, 5 μg/kg daily, starting on days 5–7 can be used until the absolute neutrophil count rises above 1.5 × 10^9^/L.

### 5.3 Radiotherapy

The asco guidelines 2 indicate that csfs should be avoided in patients receiving chemotherapy and concomitant radiation, particularly radiation involving the mediastinum. Therapeutic use of csfs may be considered in patients receiving radiotherapy alone if prolonged delays secondary to neutropenia are expected. In practice, csfs are not generally used in radiotherapy because of the lack of evidence to suggest an improvement in the rate of complication or survival. In Canada, csfs are not approved for use with radiotherapy.

## 6. DOSING AND FORMULATION OF CSFs

Currently two formulations of g-csf are approved for use in Canadian clinical practice. Filgrastim (r-methug-csf) stimulates the production of neutrophil precursors, enhances the function of mature neutrophils, and reduces the duration of neutropenia (and thus its complications). Filgrastim is cleared by the kidneys, and so its plasma half-life is 3–4 hours. Daily administration of the drug is therefore required. With the covalent binding of polyethylene glycol to the N terminus of filgrastim (producing pegfilgrastim), the plasma half-life of the drug is increased such that pegfilgrastim levels as a function of the neutrophil count become “self-regulating” 47. The net result is that a single injection of pegfilgrastim is equivalent to multiple daily injections of filgrastim.

Two large randomized controlled trials compared single administration of pegfilgrastim with daily filgrastim in patients receiving myelosuppressive chemotherapy (an anthracycline–taxane regimen) 48,49. The larger of the two trials randomized 310 breast cancer patients to either a single subcutaneous injection of pegfilgrastim 100 μg/kg on day 2 or to daily subcutaneous injections of filgrastim at 5 μg/kg beginning on day 2 and continuing until the anc was documented at 10 × 10^9^/L or higher after the expected nadir or for up to 14 days, whichever occurred first 48. The second study randomized 157 patients in an identical design, except that a fixed dose of 6 mg of subcutaneous pegfilgrastim was used 49. The dose and duration of the filgrastim in the standard arms was identical across both studies. Both studies demonstrated that pegfilgrastim was safe and well tolerated, as filgrastim was. In regard to duration of severe neutropenia and the depth of the anc nadir, the effects of pegfilgrastim were similar to those of filgrastim. However, in one study, the incidence of fn was significantly lower in the pegfilgrastim arm 48.

Pegfilgrastim and filgrastim both offer significant and similar benefits following moderate-to-severe myelosuppressive chemotherapy for the treatment of cancer. The additional advantages of pegfilgrastim include the single injection (convenience for patient and health care provider) and also potentially a lower rate of fn. Both formulations of g-csf should be considered for patients with solid tumours or lymphomas requiring a csf for primary or secondary prophylaxis.

## 7. DURATION OF THERAPY WITH CSFs

As demonstrated in the studies mentioned in the previous subsection, and in the many studies contributing to a recent systematic review of primary prophylaxis with g-csf 12, the use of filgrastim should be initiated soon after delivery of chemotherapy (most studies started on day 2) and continued until a documented post-nadir anc recovery to 1.5 × 10^9^/L or higher is reached. The key goal is to continue until after the expected nadir. The exact anc that it is clinically important to achieve is debatable; 1.0–1.5 × 10^9^/L or higher is suggested. Unless daily blood counts are being monitored, a conservative approach, ensuring that the anc rises well above the desired level, is wise. Often, between the last filgrastim dose and day 1 of the subsequent cycle of chemotherapy, a significant gap occurs during which the anc drops to some degree. In the study that investigated subcutaneous pegfilgrastim 49, the median time to anc recovery to 2.0 × 10^9^/L or higher with anthracycline– taxane chemotherapy was 9 days from the day of chemotherapy.

The duration of filgrastim therapy will also depend on the time to anc nadir and the duration of grade 4 neutropenia. Therefore, one additional advantage of pegfilgrastim is its “self-regulation” with a single dose, obviating the need for blood count monitoring and significantly reducing the risk of overshooting the target. Daily administration of filgrastim is currently indicated, although other schedules have been tested. Data from a nonrandomized observational study published by Papaldo *et al.*^50^ showed that a less frequent g-csf dosing schedule was associated with a benefit equivalent to that of daily administration in women undergoing adjuvant chemotherapy for breast cancer, although the rate of fn in the control arm was only 7%.

## 8. DISEASE-SPECIFIC RECOMMENDATIONS

Given the prevalence and incidence of breast cancer, lymphoma, and gastrointestinal and lung cancers, this subsection presents a more focused analysis of the available data on fn prevention and the use of csfs in those specific diseases.

### 8.1 Breast Cancer

Significant advances have been made in adjuvant systemic therapy for early-stage breast cancer. Those advances include the use of anthracyclines, the advent and implementation of dose-dense chemotherapy, and more recently, the addition of taxanes. Although all of the foregoing therapeutic approaches have resulted in improved patient outcomes 51–56, it is important to recognize the related toxicities and to ensure that appropriate supportive care measures are taken to mitigate the effects of those toxicities.

Table II summarizes the adjuvant breast cancer regimens commonly in use in Canada and the associated rates of fn. These data indicate that most of the adjuvant protocols have fn rates under 10%, but that some protocols would have allowed for secondary csf prophylaxis. Notably, however, clinical trial populations tend to be healthier than the general population with the same diagnosis, which means that the rates of fn reported in clinical trials may be lower than those seen in clinical practice. The two adjuvant protocols in which primary prophylaxis is definitely recommended are dose-dense cyclophosphamide–doxorubicin (ac) followed by paclitaxel, and docetaxel, doxorubicin, and cyclophosphamide (tac) 6,55. Primary prophylaxis with tac chemotherapy reduces the fn rate to 7.5% from 28.8% 56.

Recent data have showed that fec followed by docetaxel is superior to fec100 given for 6 cycles and has a fn rate of 11.2% 54. That finding has led to wide adoption of the fec protocol in Canada. Patients who are being considered for fec treatment followed by docetaxel should be carefully assessed. Based on current guidelines, primary prophylaxis should be considered for patients with significant risk factors for fn.

Breast cancer treatment regimens continue to evolve, and new treatments are being developed. Tools are also now available to determine which patients are likely to benefit from chemotherapy. In the future, the best way to reduce chemotherapy-associated toxicities may be to sequester the patients who will not benefit from chemotherapy.

### 8.2 Lymphoma

Aggressive-histology nhl, such as diffuse large B cell, represent potentially curable neoplasms, even in older adults. In a pivotal randomized trial, chop chemotherapy (cyclophosphamide–doxorubicin– vincristine–prednisone) was shown to be as effective as, and less toxic than, more complex second-and third-generation regimens 57. Since then, the addition of rituximab to chemotherapy in patients with aggressive-histology *CD20-*positive lymphoma has improved outcomes in both older 58 and younger patients 59. Additional studies have demonstrated the potential benefits of dose-dense chemotherapy supported by primary prophylaxis in older adults 7 (that is, chop given on a 14-day schedule as compared with a 21-day schedule), but final publication of the results of dose-dense chemotherapy with rituximab (chop-r) are awaited 60–62. In Canada, chop-r has been the standard regimen for aggressive-histology nhl that expresses *CD20.* Administration of chop-r could be associated with a fn rate of 10% or less 59 or in the 10% to 20% range 58, but the rate could be much higher in elderly patients or in those with comorbidities or poor performance status 63,64.

Many nhl patients are older and therefore at increased risk for chemotherapy-related toxicities 63,64, particularly infectious and hematologic toxicities. Several clinical trials have demonstrated that a combination of chop-like chemotherapy with rituximab or dose-dense chop can improve outcomes for older adults with aggressive-histology B cell nhl 58,65,66.

Providing chemotherapy on an accepted schedule has become the standard of care for patients with potential curable aggressive-histology nhl. Although no prospective randomized studies have tested standard against less-than-standard dis, results of published studies have suggested that maintaining the di of chemotherapy in aggressive nhl is important 67–70. Furthermore, regimens that were designed to be less toxic than standard chop have produced inferior outcomes in older adults with nhl 66,71,72.

Current international guidelines suggest primary prophylaxis with g-csf for all older patients (typically 65 years of age and older) with aggressive-histology lymphoma who are receiving curative-style (chop-r–like) chemotherapy 2,5. Given the importance of maintaining di, secondary prophylaxis is also valuable in patients of any age who are being treated for nhl with curative intent.

### 8.3 Gastrointestinal Cancer

Colorectal cancer is the second leading cause of cancer death in Western countries 73, and 50% of patients who undergo surgery alone for cure ultimately relapse and die of their disease 74. In 2002, results of the mosaic trial were reported at the asco annual meeting. With the use of folfox (5-fu–leucovorin–oxaliplatin) infusional therapy, 2% of patients relapsed or died as compared with 26% in the 5-fu–leucovorin arms. This improvement in survival was associated with a 41% rate of grades 3 and 4 neutropenia in patients receiving oxaliplatin, but the neutropenia was complicated by fever or infection in only 1.8% of patients. Adjuvant therapy with 5-fu–leucovorin produced only a 4.7% rate of grades 3 and 4 neutropenia, and only a 0.2% rate of associated fever 75. The recently revised asco guidelines 2 for the use of csfs in patients with a greater-than-20% risk of fn currently preclude the use of those agents prophylactically.

### 8.4 Lung Cancer

The hematologic toxicities of the various chemotherapy regimens in patients with sclc and non-small-cell lung cancer (nsclc) were included in the eortc 3 guideline summary tables (see that guideline’s Table 4) and in the American Society of Hematology/ asco guideline 2 (see that guideline’s Table 1). Depending on the characteristics of the lung cancer subtype and the regimen selected, fn rates in excess of 20% may be seen. For patients with nsclc, few data are available demonstrating any benefit in response rate or survival from primary prophylaxis with a g-csf 76. A meta-analysis of randomized trials evaluated the role of csfs in patients with sclc both for maintaining and for increasing di 22. In the seven studies designed to maintain di, the response rate was higher in the groups that received csfs (rr: 0.92; 95% ci: 0.87 to 0.97), but os was not better [hazard ratio (hr): 1.0; 95% ci: 0.94 to 1.13]. In five trials in which csfs were used to increase di, no detectable increase was observed in either response rate (rr: 1.02; 95% ci: 0.94 to 1.09) or os (hr: 0.82; 95% ci: 0.67 to 1.0). In a more recently published randomized study 77 of g-csf prophylaxis in 175 patients with sclc who were treated with cyclophosphamide, doxorubicin, and etoposide, and who were all given prophylactic antibiotics, g-csf reduced the incidence of fn to 18% from 32% (rr: 0.57; 95% ci: 0.34 to 0.97). The difference in the rate of fn in the first cycle was 24% as compared with 10% (*p* = 0.01), indicating an early benefit for treatment with g-csf, despite the use of prophylactic antibiotics.

## 9. SAFETY

Differences in the chemical structures of the csfs have produced various therapeutic agents. Filgrastim (Neupogen) is identical to endogenous g-csf except that it has an added N-terminal methionine. Pegfilgrastim (Neulasta) has a polyethylene glycol molecule bound to the N-terminal methionine; this structural difference imparts a different pharmacokinetic profile. Lenograstim (Granocyte: Chugai Pharmaceutical, Bedminster, NJ, U.S.A.) is a glycosylated product identical to the endogenous human molecule 78. Despite their chemical differences, all of these molecules interact with the g-csf receptor and initiate downstream signalling through the jak–stat (Janus kinase–signal transducers and activators of transcription) intracellular pathway 79, thus enhancing the activity, production, and release of neutrophils into the peripheral blood.

With the expanded use of csfs comes a growing body of data and literature concerning safety and associated toxicities. The toxicities review that follows focuses on post-chemotherapy toxicities. Data are supplemented with the recorded toxicities for csf use in the treatment of myelodysplasia and the procurement of stem cells in peripheral blood collection.

### 9.1 Acute Toxicity

The short-term side effects of csfs are generally mild and seldom require dose adjustments or drug cessation. Documented acute toxicities include bone pain (25%–30%), headache (16%–55%), fatigue (6%– 33%), nausea (3%–18%), myalgia (5%–41%), insomnia (6%–30%), fever (2%–27%), and anorexia (11%) 80. A multivariate analysis performed by Murata *et al.*^81^ on apheresis donors indicated that g-csf given at doses higher than 8 μg/kg daily was associated with increased bone pain; headache was more frequent in donors younger than 35 years of age; and nausea or vomiting (or both) were more frequent in female donors. Most acute toxicities of csfs can be controlled with conservative measures and non-opioid or opioid analgesics. The administration of dexamethasone did not seem to ameliorate g-csf– related adverse events 82. Astemizole, an oral antihistamine, has been reported to reduce g-csf–induced bone pain unresponsive to acetaminophen 83.

Self-limiting laboratory abnormalities, including elevated alkaline phosphatase, lactate dehydrogenase, uric acid, alanine aminotransferase, and gamma-glutamyl transpeptidase, and decreased potassium and magnesium have also been reported. Although laboratory coagulation abnormalities have been noted in the literature 84, clinical thrombotic sequelae are rare and do not suggest induction of a frank hypercoagulable state.

The potential for csfs to induce anemia has been investigated. Papaldo *et al.*^85^ evaluated an adjuvant anthracycline regimen with or without g-csf in early breast cancer. In a recent exploratory hypothesis-generating analysis of that trial, the use of g-csf was associated with a higher incidence of grade 2 anemia (38.8% vs. 26.2%, *p* = 0.005) even though the chemotherapy di did not differ between the two study arms 86. Nonetheless, no difference in clinical outcomes (such as the need for red blood cell transfusion) was detected between the study arms.

Three trials compared pegfilgrastim with filgrastim in the setting of prophylactic csf support following chemotherapy 48,49,87 and showed similar acute toxicity profiles.

### 9.2 Cutaneous Toxicity

Skin toxicities from csfs can be categorized into three patterns:

- Injection site reactions are the most common, with one case series reporting a 25% incidence of localized painful or pruritic wheals 88.
- Generalized *de novo* skin toxicities are rare, but reports of Sweet syndrome, bullous pyoderma gangrenosum, leukocytoclastic vasculitis, and folliculitis have all been published 89.
- Isolated cases of csfs exacerbating pre-existing cutaneous inflammatory disorders such as vasculitis and psoriasis have also been documented.

### 9.3 Pulmonary Toxicity

Anecdotal accounts of csf-induced pulmonary toxicity have been published, including cough, dyspnea, pulmonary infiltrates 90, and acute respiratory distress syndrome 91, which is thought to be mediated by neutrophil- induced alveolar capillary wall damage 92, although such reports are exceedingly rare.

In 2001, a systematic review of all published cases of csf-related pulmonary toxicity uncovered 84 cases 93. These cases were further classified into three groups:

- Pulmonary toxicity associated with csf use alone (group 1, *n* = 2)
- Pulmonary toxicity with csf used in combination with other potentially pulmonotoxic agents (group 2, *n* = 73)
- Pulmonary toxicity during csf-enhanced neutropenia recovery (group 3, *n* = 9)

The authors concluded that the evidence was insufficient to categorically link the use of csfs with significant pulmonary toxicity, because only 2 of the reported cases were directly linked to csf use. However, they did argue that csfs may interact with other potentially pulmonotoxic drugs, especially in neutropenic patients with pulmonary infiltrates, warranting close observation in that patient population.

### 9.4 Leukemogenicity

The leukemogenic potential of alkylating agents has been well established in the cancer literature 94.

The National Surgical Adjuvant Breast and Bowel Project (nsabp) experience of adjuvant, standard- dose, ac therapy in 4483 women with breast cancer revealed an 8-year incidence of treatment-induced leukemia of 0.27% 95. Despite the rarity of secondary hematologic malignancies, the newer regimens enabled by csfs may demonstrate an increase in the risk of secondary myelodysplastic syndrome (mds) and acute myelogenous leukemia (aml) because patients receive higher doses of genotoxic drugs. The better outcomes that result allow for longer survival, during which secondary hematologic malignancies may develop.

More recently, Patt *et al.*^96^ evaluated 64,715 patients from the Surveillance Epidemiology and End Results (seer)–Medicare database and demonstrated that the adjusted hr for developing aml was 1.53 (95% ci: 1.14 to 2.06) in patients who received adjuvant chemotherapy as compared with those who did not. The use of g-csfs within the 1st year of breast cancer diagnosis was not associated with an increased risk for developing aml (hr: 1.14; 95% ci: 0.67 to 1.92).

Preclinical models have suggested a possible leukemogenic effect of g-csfs 97,98; however, to date, clinical data have not confirmed it.

In the prospective randomized phase iii adjuvant breast trial by the Cancer and Leukemia Group B, Citron *et al.*^6^ compared a dose-dense regimen of chemotherapy supported by g-csf with the standard regimen. In an updated report after a median follow-up of 69 months 99, the incidence of aml or mds or a combination was no higher in the dose-dense arms than in the standard arms without routine csf support (0.70%). Other adjuvant breast cancer trials incorporating routine csfs have also failed to reveal a significantly increased risk of secondary leukemia 50,100. Trials investigating csf protocols for other disease sites (sclc, urothelial cancer, and sarcoma, for instance) have not reported the incidence of aml or mds 101–103.

The magnitude of the additional risk of csfs, if present, to the incidence of treatment-related aml or mds may be outweighed by the benefit. Population-based data and meta-analyses have attempted to elucidate an answer to that question.

In a retrospective cohort study based on seer claims data, Hershman *et al.*^104^ assessed women older than 65 years of age who received adjuvant chemotherapy, with or without csfs, for stages i–iii breast cancer between 1991 and 1999. Of the studied women, 1.16% developed aml or mds 18 months or more after diagnosis. Of the 906 patients treated with g-csfs, 16 (1.77%) developed aml or mds; of the 4604 patients not treated with g-csf, 48 (1.04%) developed aml or mds. The risk of aml or mds did not change substantially when clinical, treatment, and demographic variables were accounted for (hr: 2.14; 95% ci: 1.12 to 4.08).

This seer database analysis is based on a large numbers of patients, but as noted in an accompanying editorial 105, the data are derived from non-validated health care claims that do not provide specific data on cumulative dose or duration of either g-csf or chemotherapy. A study of this kind could equally underestimate the incidence of treatment-related malignancy in patients who died from breast cancer in the first years of follow-up. The more dose-intensive the adjuvant therapy regimen, the higher the risk of secondary leukemia. Also, failure to recover marrow after exposure to chemotherapy is an indication for g-csf, but such failure may also be a marker of marrow deficiency that may increase susceptibility to malignant transformation.

Based on the analysis by Hershman *et al.,* the use of g-csf was associated with a doubling of the risk for subsequent aml or mds in the studied population, even though the absolute risk remained low. The authors themselves suggested that even if the association were to be confirmed, the benefits of g-csf may still outweigh its risks 104.

A similar analysis was performed on a population of 182 French women who developed leukemia after adjuvant chemotherapy for early breast cancer 106. Patients who received g-csf (8.8% of the group) had a significantly increased risk of aml or mds (rr: 6.26; 95% ci: 1.89 to 20.7), although the reason for g-csf treatment, the dose, and the duration were not systematically recorded in medical files. The authors also noted that g-csf was administered chiefly because of poor hematologic tolerance to chemotherapy, which could reflect chemotherapy drug accumulation as a result of altered pharmacokinetics, metabolism, or bone marrow sensitivity of the patients.

Smith *et al.*^95^ retrospectively reviewed data from six nsabp trials that were distinguished by differences in cyclophosphamide intensity and dose, and by the presence or absence of mandated prophylactic support with growth factors. As compared with patients receiving standard chemotherapy, patients receiving dose-intense chemotherapy with g-csf support showed cumulative incidences of mds and aml of 1.01% (95% ci: 0.63% to 1.62%) and 0.21% (95% ci: 0.11% to 0.41%) respectively at 5 years. Those results should be interpreted with caution, because increasing the chemotherapy dose is, in itself, a risk factor for aml and mds 107 and distinguishing the leukemogenicity of intensified therapy from that of g-csf administration is often difficult.

In addition to the combined analysis, Smith *et al.*^95^ also attempted to analyze the final results of nsabp B-25, a trial in which women were randomly assigned to 4 cycles of ac chemotherapy with double the cumulative dose of cyclophosphamide 108. Although use of g-csf was mandated for all patients, total g-csf dose varied considerably across patients. Controlling for treatment arm, patient age, and surgical procedure, the estimated risk for aml and mds in patients receiving more than the median dose of g-csf was 3.58 relative to patients receiving the median dose or less (*p* = 0.02). However, the authors also noted that the result was not based on a randomized comparison and that the use of g-csf was likely correlated with other factors. Also, patients achieving an unusually high plasma level of doxorubicin or cyclophosphamide, or both, are possibly at higher risk simultaneously for aml or mds and for fn and severe infection. In that case, any suggested association between the use of g-csf and the subsequent incidence of aml or mds may have no causal basis.

Long-term data relating to csf use in hematology have revealed uncertain associations with secondary aml and mds. Data from the Severe Chronic Neutropenia International Registry revealed an association between the use of csfs and acquired cytogenetic clonal abnormalities of the marrow. However, no evidence definitively related the dose of g-csf or the duration of g-csf therapy to clinical malignant transformation 109. Thus far, registry studies have not identified an increased risk of malignancy among healthy individuals who received g-csf before harvesting of stem cells from peripheral blood; however, the numbers are small, and more than 2000 donors would have to be followed for 10 years to detect a rise by a factor of 10 in the leukemia risk 110.

Finally, in a retrospective review of children undergoing chemotherapy for all at a single institution, the cumulative incidence of therapy-associated aml was significantly higher in the cohort who received g-csf in their treatment protocol than in the cohort that did not 111.

The data from the dose-dense trials and hematology reports are not conclusive. Mitigating factors such as chemotherapy dose and inherited predispositions to secondary cancers have not been fully explored. The ambiguity demands further research with longer follow-up. Clinical patterns of secondary leukemia are emerging with corresponding molecular profiles 112, thus enabling more precise definition of iatrogenic as compared with sporadic leukemia.

### 9.5 Safety Conclusions

A review of the reported toxicity data associated with csfs reveals an acceptable pattern of short-term toxicities, manageable with conservative measures alone. Further follow-up is necessary to elucidate potential associations between csfs and pulmonary toxicities, and csfs and secondary hematologic malignancies. Colony-stimulating factors should always be used within the context of approved guidelines and labelling.

## 10. SUMMARY

Neutropenia is a common complication of chemotherapy that can result in severe sequelae in cancer patients. The management of neutropenia requires a patient-specific approach, accounting for the malignancy, the chemotherapeutic regimen, and patient risk factors such as age, comorbid illness, and past history. The appropriate use of csfs is critical to managing these patients in situations of both primary and secondary prophylaxis, especially in high-risk situations in which chemotherapeutic regimens are associated with a 20% or higher risk of fn. Benefits include fewer infections, shorter hospitalizations, and possibly lesser mortality. As in all aspects of cancer care, the risks must be weighed against the benefits, tailoring the treatment to each individual patient.

## Figures and Tables

**TABLE I:** Current guidelines for primary prophylaxis with granulocyte colony–stimulating factor (g-csf) 2–4

Neutropenic event risk	ASCO 2006 2	EORTC 2006 3	NCCN 2006^4^
Moderate to high	Use G-CSF (~20%)	Use G-CSF (≥20%)	Use G-CSF (>20%)
Intermediate	Recommend G-CSF (<20%)	Consider G-CSF (10%–20%)	Consider G-CSF (10%–20%)
Low	Not specified	G-CSF not recommended (<10%)	G-CSF not recommended for most patients (<10%)
Risk factor assessment	+++	+++	++

asco = American Society of Clinical Oncology; eortc = European Organization for Research and Treatment of Cancer; nccn = National Comprehensive Cancer Network.

**TABLE II:** Common adjuvant breast cancer regimens and associated rates of febrile neutropenia (fn)

Chemotherapy regimen	FN incidence (%)
Docetaxel, doxorubicin, cyclophosphamide 55	28.8
5-Fluorouracil, epirubicin, cyclophosphamide, docetaxel 54	11.2
Oral cyclophosphamide, epirubicin, 5-fluorouracil 51	9.0
5-Fluorouracil, epirubicin, cyclophosphamide 54	8.4
Docetaxel, doxorubicin, cyclophosphamide 56,a	7.5
Docetaxel, cyclophosphamide 53	5.0
5-Fluorouracil, doxorubicin, cyclophosphamide 55	4.4
Doxorubicin, cyclophosphamide, paclitaxel 6,52	3–6
Doxorubicin, cyclophosphamide 52	0–2.5
Dose-dense doxorubicin, cyclophosphamide, paclitaxel 56,a	2.0
Oral cyclophosphamide, methotrexate, 5-fluorouracil 51	1.0

a Necessitated primary prophylaxis with granulocyte colony–stimulating factor.
